# Manganese-mediated acceleration of age-related hearing loss in mice

**DOI:** 10.1038/srep36306

**Published:** 2016-11-08

**Authors:** Nobutaka Ohgami, Ichiro Yajima, Machiko Iida, Xiang Li, Reina Oshino, Mayuko Y. Kumasaka, Masashi Kato

**Affiliations:** 1 Department of Occupational and Environmental Health, Nagoya University Graduate School of Medicine, Nagoya, Japan; 2 Nutritional Health Science Research Center, Chubu University, 1200 Matsumoto, Kasugai, Aichi 487-8501, Japan; 3 Voluntary Body for International Health Care in Universities, Nagoya, Japan

## Abstract

Despite the fact that manganese (Mn) is known to be a neurotoxic element relevant to age-related disorders, the risk of oral exposure to Mn for age-related hearing loss remains unclear. In this study, we orally exposed wild-type young adult mice to Mn (Mn-exposed WT-mice) at 1.65 and 16.50 mg/L for 4 weeks. Mn-exposed WT-mice showed acceleration of age-related hearing loss. Mn-exposed WT-mice had neurodegeneration of spiral ganglion neurons (SGNs) with increased number of lipofuscin granules. Mn-exposed WT-mice also had increased hypoxia-inducible factor-1 alpha (Hif-1α) protein with less hydroxylation at proline 564 and decreased c-Ret protein in SGNs. Mn-mediated acceleration of age-related hearing loss involving neurodegeneration of SGNs was rescued in *RET*-transgenic mice carrying constitutively activated *RET*. Thus, oral exposure to Mn accelerates age-related hearing loss in mice with Ret-mediated neurodegeneration of SGNs.

Hearing loss is one of the sensory diseases that have a negative impact on the quality of life (QOL). It is estimated that about 360 million people in the world suffer from hearing loss and about one third of people over the age of 65 years suffer from age-related (late onset) hearing loss^1^. Genetic and aging factors have been shown to cause onset of hearing loss in humans and mice based on evaluations by pure tone audiometry (PTA) and auditory brainstem response (ABR)^2^,^3^. On the other hand, previous studies indicated risks of hearing impairments in young adult humans exposed to environmental factors including noise and heavy metals^4^,^5^,^6^. In an experimental study, exposure to audible noise at excessive levels was shown to cause acceleration of age-related hearing loss in young adult mice at 4–8 weeks of age^7^. Thus, young adults have a potential risk of age-related hearing loss, though there is limited information about environmental factors other than exposure to noise that accelerate age-related hearing loss in young adult humans and mice.

Manganese (Mn) is known to be a neurotoxic element relevant to age-related disorders. In humans, excessive exposure to Mn via inhalation causes neurodegeneration of the substantia nigra that has pathological similarities to Parkinsonism^8^,^9^. High levels of Mn up to 34,000 μg/L in well drinking water have been reported in various developing countries^10^,^11^,^12^,^13^,^14^. Excessive exposure to Mn by drinking well water is also a risk for neural diseases^10^,^15^. In experimental studies, exposure to Mn by inhalation resulted in Parkinsonism in mice^16^. C57BL/6 mice (4–5 months old) orally exposed to Mn at 400 mg/L via drinking water for 5–6 weeks showed increased Mn levels in the brain including the substantia nigra, resulting in neurobehavioral defects^17^,^18^. Oral exposure to Mn also had effects on brain dopamine levels and neurocognitive functions in neonatal rats^19^. On the other hand, C57BL/6 mice are known to suffer from acceleration of age-related hearing loss at a high-frequency sound (20–40 kHz) from 4 months of age^20^. Subcutaneous injection of a high concentration of Mn (100 mg/kg) into C57BL/6 mice was shown to increase Mn levels in the inner ears^21^. The inner ears contain the organ of Corti and stria vascularis (SV). The SV maintains endolymph potential. The organ of Corti, which contains inner hair cells (IHCs) and outer hair cells (OHCs), plays an important role in conversion of sound stimulations to neural impulses, followed by transmission to spiral ganglion neurons (SGNs), which serve as the primary carrier of auditory information^22^,^23^. In a previous study, *ex vivo* exposure of SGNs to Mn at levels as low as 50 μM (6.29 mg/L) was shown to cause neurodegeneration of SGNs^24^. However, there has been no study showing an association between oral exposure to Mn via drinking water and hearing loss as well as onset of age-related hearing loss determined by ABR in experimental animals.

c-Ret is a tyrosine kinase and a receptor for neurotrophic factors including glial cell line-derived neurotrophic factor (GDNF)^25^. In our previous studies, impairment of c-RET was shown to be involved in hearing loss in humans^26^. The phoshorylation level of tyrosine 1062 (Y1062) in c-Ret directly affects kinase activity of c-Ret^25^. Partial impairment of Y1062 phoshorylation in c-Ret was shown to accelerate age-related hearing loss with neurodegenration of SGNs with impairment of NF-κB, which is a downstream molecule of c-Ret^26^ in the inner ears of *c-Ret*-knock-in mice^20^. Thus, c-Ret kinase is an age-related hearing loss-related molecule. On the other hand, hypoxia-inducible factor-1 alpha (HIF-1α) is known to be a transcriptional factor and to form a heterodimer with HIF-1β under the condition of environmental stress. A previous study showed that age-related hearing loss is associated to HIF-1α protein in SGNs^27^. Exposure to transitional elements including cobalt and Mn has been shown to stabilize HIF-1α protein with less hydroxylation at proline 564 *in vitro*^28^,^29^. Furthermore, a previous *in vitro* study showed that stabilization of HIF-1α protein is required for decrease of RET protein in neural cells exposed to cobalt^30^. Thus, the results of previous studies raise the possibility that exposure to Mn affects the onset of age-related hearing loss caused by impairment of c-Ret via HIF-1α in SGNs, though it remains unknown whether there is a correlation between HIF-1α protein and c-Ret in SGNs.

We therefore performed an experimental study to examine the correlation between Mn and age-related hearing level in humans and to clarify the mechanism of age-related hearing loss in mice exposed to Mn at possible levels ingested from drinking water.

## Results

### Oral exposure of young adult mice to Mn accelerated age-related hearing loss

**Figure 1:**
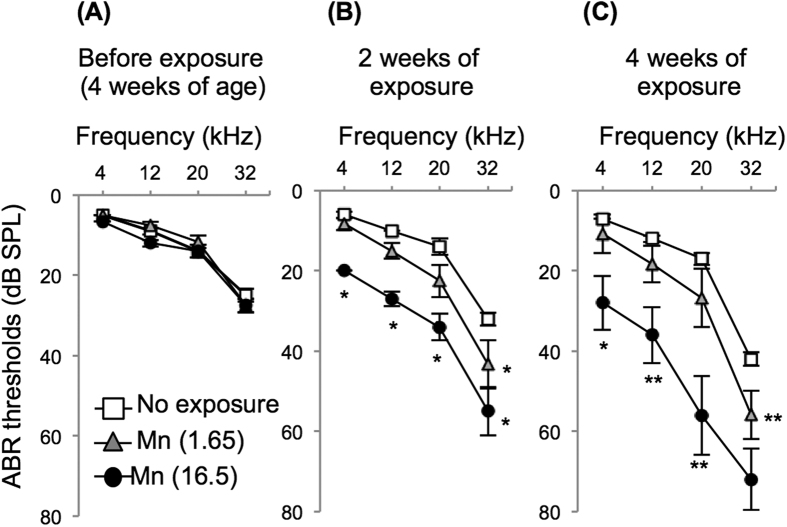
WT mice exposed to Mn showed acceleration of age-related hearing loss. (**A–C**) Hearing levels (means ± SE) of mice before exposure (**A**) and at 2 weeks (**B**) and 4 weeks after exposure (**C**) to Mn at 1.65 mg/L [Mn (1.65), gray triangles; n = 6] and at 16.50 mg/L [Mn (16.50), black circles; n = 6] and of mice in the non-exposure group (open squares; n = 10). Significant differences (*p < 0.05, **p < 0.01) were analyzed by the Steel-Dwass test.

We performed an experimental study with wild-type young adult C57BL/6J mice (WT mice) at 1 month of age exposed to Mn at 1.65 and 16.50 mg/L for 4 weeks. Before exposure, hearing levels in all groups were comparable (Fig. 1A). The non-exposure group (n = 10) showed age-related hearing loss at a high-frequency sound (32 kHz) (Fig. 1C), corresponding to results of previous studies^20^,^31^. WT mice exposed to Mn at 1.65 mg/L showed an increased threshold of ABR at 32 kHz compared with that in the non-exposure group (Fig. 1B,C). WT mice exposed to Mn at 16.50 mg/L for 2 weeks and 4 weeks showed severe hearing loss at 1–32 kHz compared to the hearing level in the non-exposure group (Fig. 1B,C).

### Mn-mediated acceleration of age-related hearing loss in mice involved neurodegeneration of SGNs

**Figure 2:**
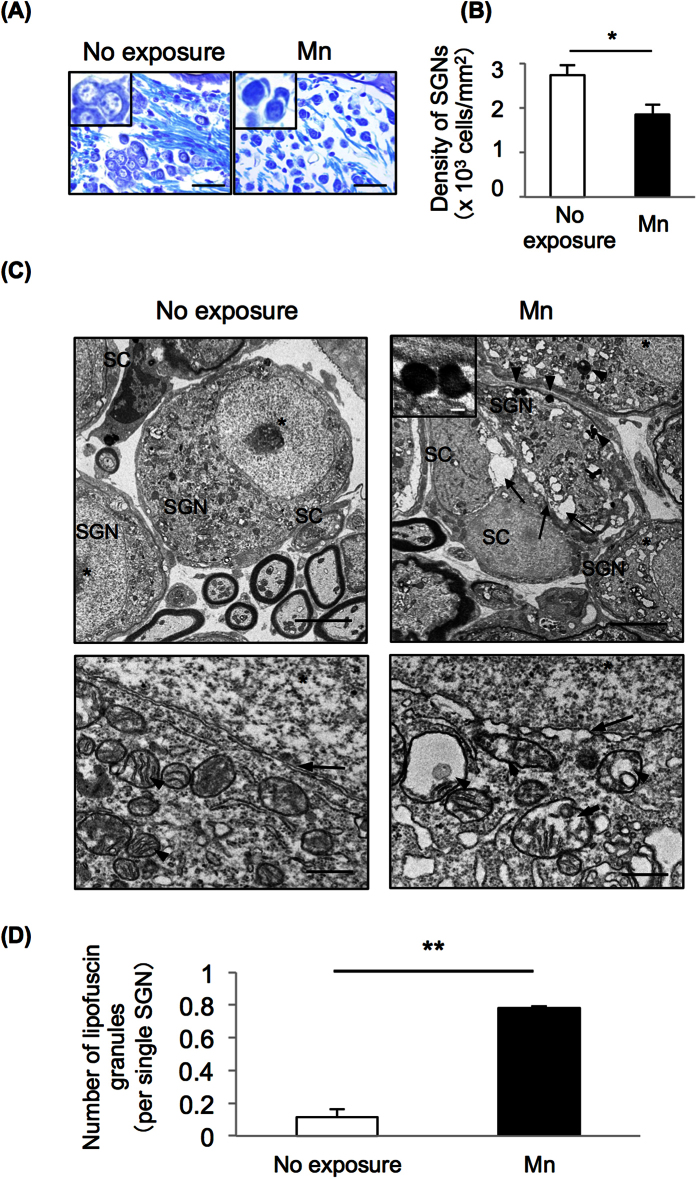
Oral exposure of WT mice to Mn caused neurodegeneration in SGNs. (**A**) Nissl staining of SGNs in the mid turn from mice administered Mn at 16.50 mg/L (Mn; right panels) and non-exposed mice (left panels). The inset in the left panel shows a typical example of SGNs with normal morphology in the non-exposure group, while the inset in the right panel shows an impaired staining pattern of nuclei of SGNs in the Mn exposure group. Scale bars: 20 μm. (**B**) Densities of SGNs (mean ± SD) without an impaired staining pattern of nuclei in the mid turn from WT mice exposed to Mn (Mn, black bars, n = 3) and non-exposed mice (white bars, n = 3) are presented. Significant difference (*p < 0.05) from the control mice was analyzed by the unpaired t-test. (**C,D**) Transmission electron microscopy (TEM) for SGNs in the mid turn from Mn-administered mice (Mn; right panels) and non-exposed mice (left panels). Asterisks indicate the nucleus. Gaps between SGNs and SCs (right upper panel, arrows) and lipofuscin granules (right upper panel, arrowheads; inset in the right upper panel) were observed in WT mice exposed to Mn. Scale bars: 5 μm (upper panels), 500 nm (lower panels) and 200 nm (inset). (**D**) The number of lipofuscin granules per single SGN (mean ± SD) was counted. Significant difference (**p < 0.01; *p < 0.05) from the control mice was analyzed by the unpaired t-test.

We then performed morphological analysis of the inner ears from WT mice exposed to Mn in order to determine the pathogenesis of age-related hearing loss caused by Mn administered by drinking water. Nissl staining revealed that WT mice exposed to Mn had a decreased density of SGNs without an impaired staining pattern of nuclei (Fig. 2A, right panel, inset and B) compared to the morphology and density of SGNs in non-exposed mice (Fig. 2A, left panel, inset and B). We further performed detailed morphological analyses of SGNs from WT mice exposed to Mn by transmission electron microscopy (TEM) (Fig. 2C). Gaps between SGNs and Schwann cells (SCs) (Fig. 2C, right upper panel, arrows), increased number of lipofuscin granules (Fig. 2C, right upper panel, arrowheads and D) and vacuole degeneration were observed in WT mice exposed to Mn (Fig. 2C, right lower panel, arrowheads), in contrast to intact cellular membranes (Fig. 2C, left upper panel) and mitochondria (Fig. 2C, left lower panel, arrowheads) in non-exposed mice. The nuclei of SGNs from WT mice exposed to Mn showed discontinuous nuclear membranes (Fig. 2C, right lower panel, arrow), in contrast to the intact nuclear membranes observed in non-exposed mice (Fig. 2C, left lower panel, arrow).

### Mn-mediated acceleration of age-related hearing loss in mice involved impairment of Hif-1α and c-Ret in SGNs

**Figure 3:**
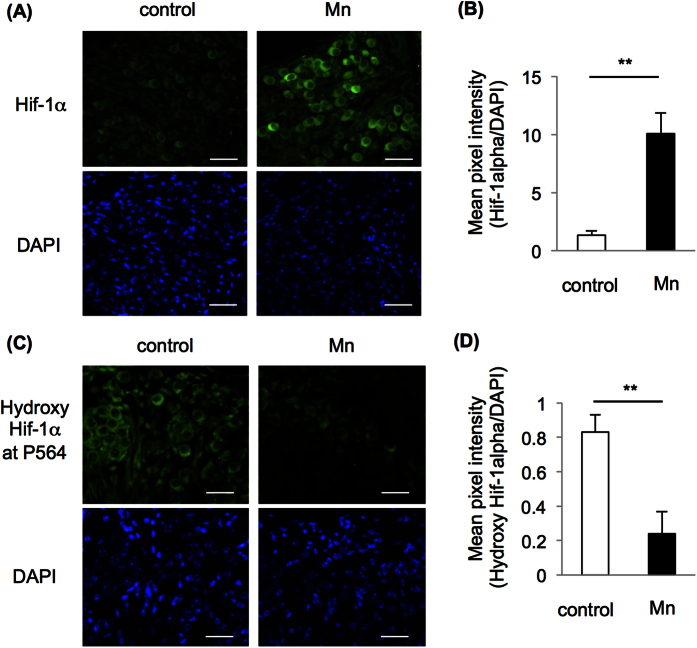
Oral exposure of WT mice to Mn increased Hif-1α protein with less hydroxylation at proline 564 in SGNs. Immunohistochemistry with anti-Hif-1α (**A**) and anti-hydroxy Hif-1α at proline 564 of SGNs in the mid turn from mice administered Mn at 16.50 mg/L (Mn; right panels) and non-exposed mice (left panels). Scale bars: 20 μm. Mean pixel intensities (means ± SD) of Hif-1α (**B**) and hydroxyl Hif-1α at proline 564 (**D**) of SGNs in the mid turn from WT mice exposed to Mn (Mn, black bars, n = 3) and non-exposed WT mice (white bars, n = 3) are presented. All positive signals were normalized by DAPI. Significant difference (*p < 0.05, **p < 0.01) from the control was analyzed by the unpaired t-test.

**Figure 4:**
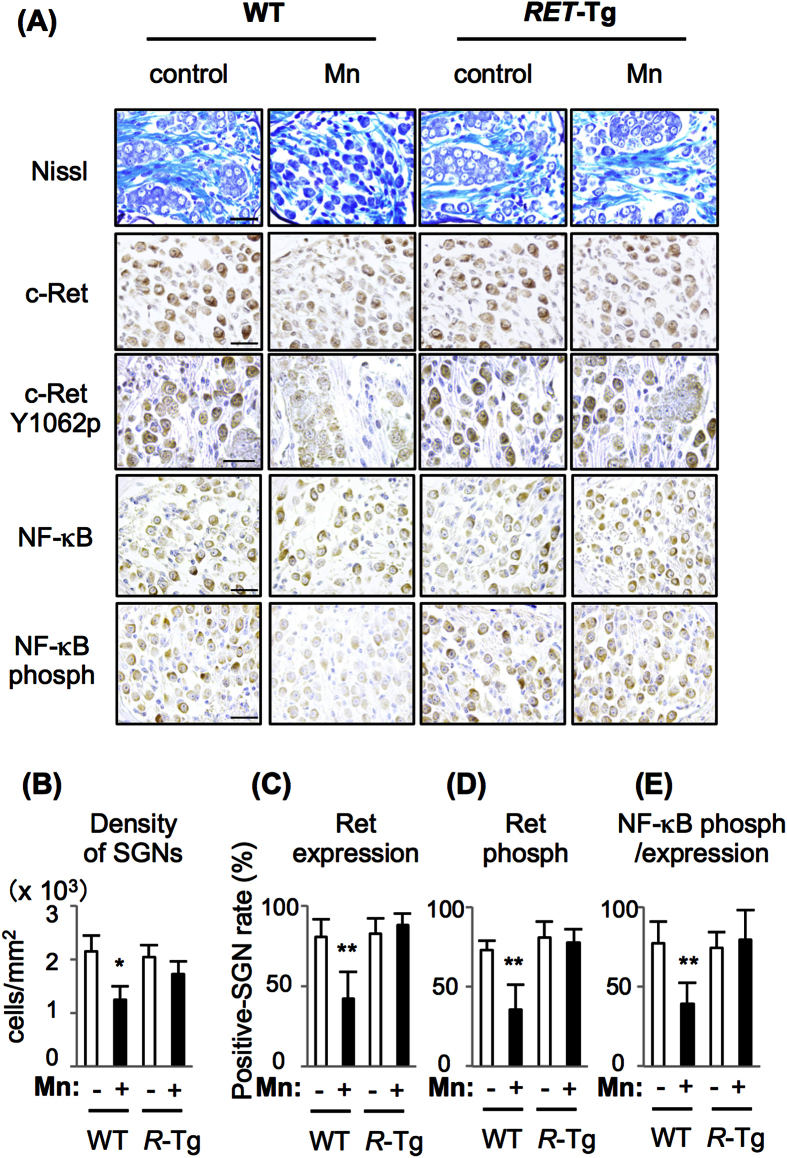
Mn-mediated impairment of c-Ret in SGNs. (**A**) Nissl staining (top panels) and results of immunohistochemical analyses with polyclonal antibodies against c-Ret, phosphorylated c-Ret at Y1062 (c-Ret Y1062p), NF-κB and phosphorylated NF-κB (NF-κB phosph) are presented. All specimens were SGNs from wild-type (WT) and littermate *RET*-Tg (*R*-Tg) mice without (control) or with exposure to Mn at 16.50 mg/L for 4 weeks (Mn). All immunohistochemical analyses were performed with diaminobenzidine followed by counterstaining with hematoxylin. Scale bars: 20 μm. (**B–D**) Densities (means ± SD) of SGNs without an impaired staining pattern of nuclei (**B**) and percentages of positive SGN numbers (means ± SE) of c-Ret expression (**C**), phosphorylated c-Ret (**D**) and phosphorylated NF-κB normalized by NF-κB expression (**E**) in the mid turn from 2-month-old WT and littermate R-Tg mice with (+) or without (−) exposure to Mn. Significant differences (*p < 0.05, **p < 0.01) were analyzed by the Tukey test.

**Figure 5:**
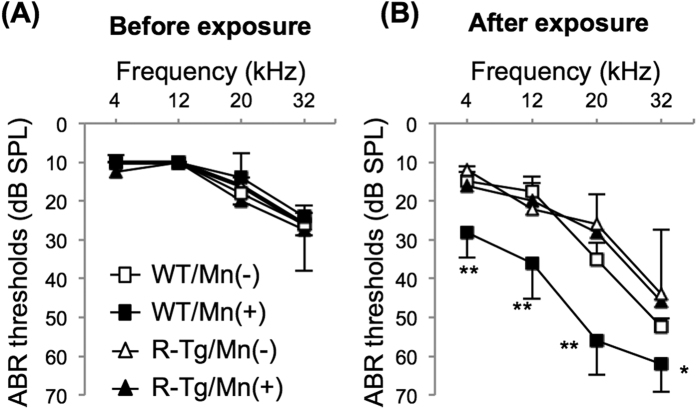
Mn-mediated age-related hearing loss in mice was rescued by introducing constitutively activated *RET*. (**A,B**) Hearing levels (means ± SE) of WT (squares) and littermate R-Tg mice (triangles) before exposure (**A**) and after exposure (**B**) with [Mn(+)] or without [Mn(−)] exposure to Mn at 16.50 mg/L for 4 weeks (n = 5, each group). Significant differences (*p < 0.05, **p < 0.01) were analyzed by the Steel-Dwass test.

We finally analyzed the mechanism of Mn-mediated hearing loss in mice. Immunofluorescent staining showed an increased level of Hif-1α and a decreased level of hydroxyl Hif-1α at proline 564 in SGNs from WT mice exposed to Mn (Fig. 3). Next, we used *RET-transgenic mice carrying constitutively activated RET (RFP-RET) (RET*-Tg mice) of line 242, in which no tumor develops without exception^20^,^26^,^32^, and litter WT mice in order to determine whether Mn-mediated hearing loss involves impairment of c-Ret in SGNs *in vivo*. WT mice exposed to Mn showed decreased levels of c-Ret protein and phosphorylation in SGNs compared to those in the non-exposed groups and *RET*-Tg mice exposed to Mn (Fig. 4A–C). WT mice exposed to Mn also showed a decreased density of SGNs without abnormal morphology, while the density and morphology of SGNs in *RET*-Tg mice exposed to Mn were comparable to those in non-exposed WT mice and non-exposed *RET*-Tg mice (Fig. 4A,D). WT mice exposed to Mn also showed decreased phosphorylation of NF-κB in SGNs compared to those in the non-exposed groups and *RET*-Tg mice exposed to Mn (Fig. 4A,E). Correspondingly, hearing levels in *RET*-Tg mice exposed to Mn were comparable to those in the non-exposed groups, while WT mice exposed to Mn showed hearing loss (Fig. 5B). All of the groups showed comparable hearing levels before exposure (Fig. 5A).

## Discussion

This study showed acceleration of age-related hearing loss in young adult mice orally exposed to Mn. Correspondingly, the WT mice orally exposed to Mn showed significantly higher levels of Mn in inner ears than those in the non-exposed mice, while the two groups of mice showed comparable Mn levels in the cerebrum, cerebellum, heart, kidney, muscle and bone (Table S1). Thus, the results suggest that oral exposure to Mn during young adulthood increases Mn levels in inner ears, resulting in acceleration of age-related hearing loss in mice.

Our results showed Mn-mediated neurodegeneration of SGNs with increased lipofuscin granules. Lipofuscin granules are known to be undegradable protein aggregates and to be involved in age-related degeneration^33^. Our results partially correspond to results of a previous study showing that age-related hearing loss involved neurodegeneration of SGNs with accumulation of lipofuscin granules in SAMP8 mice^34^, while a previous study has shown Mn-mediated aggregation of alpha-synuclein in the frontal cortex in non-human primates injected with manganese sulfate (MnSO_4_) at 3.3–10.0 mg/kg/week for maximum 52 weeks^35^. Thus, it is possible that oral exposure to Mn accelerates age-related neurodegeneration of SGNs with protein aggregates.

Morphological abnormalities in the organ of Corti including IHCs, OHCs and the SV were undetectable in WT mice exposed to Mn in this study (Figure S1). These results correspond to results of our previous studies showing that c-Ret-mediated neurodegeneration of SGNs occurred in *c-Ret*-knock-in mice, while morphological abnormalities in IHCs, OHCs and the SV were undetectable^20^,^26^. Thus, our morphological analyses suggest that Mn-mediated degeneration occurs in SGNs but not in hair cells and the SV under the exposure conditions.

Our results showed that Mn-mediated age-related hearing loss involved an increased level of Hif-1α protein with less hydroxylation at proline 564 and decreased expression and phosphorylation levels of c-Ret in SGNs. In previous studies, *in vitro* exposure to Mn increased HIF-1α protein in HepG2 cells^36^ and stabilized HIF-1α protein by direct inhibition of HIF-prolyl hydroxylase in a cell free experiment^29^. A previous study also showed that exposure to audible noise resulted in stabilization of Hif-1α protein in inner ears including SGNs^37^. Thus, it is possible that Hif-1α protein is one of the molecular targets in inner ears for environmental factors including Mn. Since stabilization of HIF-1α protein has been shown to decrease *c-RET* mRNA in a human lung cell line^38^ and to decrease c-RET protein in a human neuroblastoma cell line^30^, we hypothesized that the mechanism of Mn-mediated decrease of c-Ret protein occurs at the transcriptional level mediated by stabilization of Hif-1α protein in SGNs (Figure S2).

In conclusion, our study provides novel evidence of Mn-mediated ototoxicity showing acceleration of age-related hearing loss in young adult mice. Our results further showed that Mn-mediated age-related hearing loss as well as decreased cell density of SGNs with impairment of c-Ret and NF-κB were restored by introducing constitutively activated *RET*. Our results partially correspond to results of our previous study showing that partial impairment of c-Ret accelerated age-related hearing loss with neurodegeneration of SGNs with impairment of NF-κB in *c-Ret*-knock-in mice^20^. Thus, our results suggest the mechanism of neurodegeneration of SGNs caused by impairment of c-Ret in young adult mice orally exposed to Mn.

## Materials and Methods

### Mice

*RET*-Tg mice of line 242^32^ and littermate WT mice were previously reported. WT mice showed age-related hearing loss due to the C57BL/6 genetic background as previously reported^39^. Hearing levels in 10-week-old RET-Tg mice and littermate WT mice have been shown to be comparable^40^. All experiments were approved by the Institutional Animal Care and Use Committee in Nagoya University (approval number: 28251) and Chubu University (approval number: 2810030) and the Institutional Recombinant DNA Experiment Committee in Nagoya University (approval number: 14–88, 13–35) and Chubu University (approval number: 13–03) and followed the Japanese Government Regulations for Animal Experiments.

### Oral exposure to Mn

Manganese (II) chloride (MnCl_2_·4H_2_O) purchased from Nacalai Tesque was dissolved in distilled water. Mice were exposed to Mn at 1.65 and 16.50 mg Mn/L via drinking water. The drinking water containing Mn was freshly prepared and changed every week. The non-exposure group was given distilled water. The exposure was started at 1 month of age. We regularly monitored the amounts of drinking water and food ingested by the mice and the body weights of mice during exposure as described previously^41^. The monitoring showed that one mouse (20–30 g in body weight) consumes about 4–6 ml water per day and that mice exposed to Mn and those not exposed to Mn showed no significant difference in the intake of either food or water or in body weight. Mice were maintained under specific pathogen-free (SPF) conditions at a fixed temperature (23 ± 2 °C) and a 12-h light/dark cycle.

### Measurement of hearing

ABR measurements (AD Instruments Pty. Ltd.) were performed as described previously^20^,^26^. Tone burst stimuli were measured 5 dB-stepwise from 0 dB SPL to 90 dB SPL. The measurements were performed by researchers who were blinded to the experimental groups. The threshold was judged by an appearance of the lowest level of the I wave of ABR. Data are presented as means ± SE.

### Morphological analysis with a light microscope and a fluorescent microscope

Morphological analyses were performed as described previously^20^,^26^. The morphological analyses were performed by researchers who were blinded to the experimental groups. Nissl staining was performed with paraffin sections. Stained cells with round and palely stained nuclei were considered to be surviving cells, whereas shrunken cells with pyknotic nuclei were considered to be nonsurviving cells as described previously^42^. Immunohistochemistries with polyclonal antibodies against c-Ret protein (1:100; Immuno Biological Laboratories), phosphorylated c-Ret Y1062 (1:100; Abcam) and phosphorylated NF-κB p50 (1:50; Santa Cruz) were performed for frozen sections with Can Get Signal immunostaining solution (TOYOBO). We used a VECTASTAIN ABC Rabbit IgG kit (Vector) and 3, 3′-diaminobenzidine (DAB) kit (DAKO) to detect the primary antibodies. Specimens were observed under a light microscope (Leica DM1000LED). Immunohistochemical analysis with polyclonal antibodies against Hif-1α (1:100; Abcam), Hif-1α hydroxyl proline 564 (1:30; Novus Biologicals) and NF-κB p50 (1:100; Santa Cruz) was performed for paraffin sections. Antigen retrieval was performed with a citrate buffer (pH 6.0) at 90–92 °C for 10 minutes for the detection of Hif-1α and NF-κB p50. We used Alexa Fluor 488-labeled donkey anti-rabbit IgG (1:1000, Invitrogen) as a secondary antibody followed by counterstaining with 4′,6-diamidino-2-phenylindole (DAPI) to detect Hif-1α and Hif-1α hydroxyl proline 564 under the fluorescent microscope (Leica DMI6000B). We used the software program WinROOF (Mitani Corp., Fukui, Japan) to estimate the percentage of positive SGNs detected by antibodies as previously reported^20^,^26^.

### Morphological analysis by electron microscopy

Preparation of tissues for TEM basically followed the previous method^20^,^26^. In brief, we performed perfusion fixation with a mixture of 2% paraformaldehyde (PFA) and 2% glutaraldehyde in 0.3 M HEPES-buffer (pH 7.4) and then dissected murine cochleas. The cochleas were immersed in the same fixative solution overnight at 4 °C. The cochleas were further fixed with 2% osmium tetroxide in 0.3 M HEPES-buffer (pH 7.4) at 4 °C for 3 hours. After dehydrating the cochleas with a graded series of ethanol, we embedded the cochleas in epoxy resin (Quetol 651). We observed ultrathin sections (t = 70 nm) under an electron microscope at 80 kV (JEOL JEM1200EX, Tokyo, Japan). The number of lipofuscin granules in SGNs from the mid turn was counted. A total of 60 cells per area were counted. For scanning electron microscopy, we performed perfusion fixation with the same mixture of 2% PFA and 2% glutaraldehyde. After dissection of inner ears under a stereomicroscope, stepwise dehydration in ethanol solutions and dry up with a critical point dryer (Hitachi HCP-2) were performed. Prepared inner ears were eventually mounted on aluminum stubs with colloidal silver adhesive and sputter-coated with gold palladium before imaging by a scanning electron microscope (Hitachi S-800s).

### Statistical analysis

Statistical analyses were performed by the method previously reported^43^,^44^,^45^,^46^. We used the two-tailed Mann-Whitney *U* test (equivalent to the Wilcoxon rank sum test) and Steel-Dwass test for nonparametric data to determine a significant difference of hearing levels between two groups and among three groups, respectively, since hearing levels are discontinuous variables. We used the unpaired t-test for parametric data to determine a significant difference of the morphological analyses between two groups. We also performed one-way ANOVA followed by Tukey’s post-hoc multiple comparison tests to determine significant differences among four groups. The Steel-Dwass test was also performed with the alpha level set to 0.05. A difference with p < 0.05 was considered significant. All statistical analyses were performed using JMP Pro (version 11.0.0; SAS Institute Inc., Cary, NC, USA).

## Additional Information

**How to cite this article**: Ohgami, N. *et al*. Manganese-mediated acceleration of age-related hearing loss in mice. *Sci. Rep*. **6**, 36306; doi: 10.1038/srep36306 (2016).

**Publisher’s note:** Springer Nature remains neutral with regard to jurisdictional claims in published maps and institutional affiliations.

## Supplementary Material

Supplementary Information
