# Supplementary material for: Manganese-mediated acceleration of age-related hearing loss in mice
Source: Sci Rep. 2016 Nov 8;6:36306. doi: 10.1038/srep36306 (PMC5099889; doi:10.1038/srep36306)
Supplement: Supplementary Information [file srep36306-s1.pdf]

Manganese-mediated acceleration of age-related hearing loss in mice

Nobutaka Ohgami[1,2,3], Ichiro Yajima[1,3], Machiko Iida[1], Xiang Li[1], Reina Oshino[1,3], Mayuko Y Kumasaka[1] and Masashi Kato[1,3,*]

Author Affiliation:

[1]Department of Occupational and Environmental Health, Nagoya University Graduate School of Medicine, Nagoya, Japan.

[2]Nutritional Health Science Research Center, Chubu University, 1200 Matsumoto, Kasugai, Aichi 487-8501, Japan.

[3]Voluntary Body for International Health Care in Universities, Nagoya, Japan.

[*]**Correspondence:**

Masashi Kato M.D., Ph.D.
Department of Occupational and Environmental Health,
Nagoya University Graduate School of Medicine
Address: 65 Tsurumai-cho, Showa-ku, Nagoya, Aichi 466-8550, Japan.
Phone: +81-52-744-2122. Fax: +81-52-744-2124.
E-mail: katomasa@med.nagoya-u.ac.jp

Supplementary information

Figure legends

Figure S1. Morphological analyses of hair cells and the stria vascularis (SV) from WT-mice exposed to manganese (Mn). (*A*) Succinate dehydrogenase (SDH) histochemistry of hair cells from the group exposed to Mn at 16.50 mg/L via drinking water (n = 4) and the non-exposure group (n = 4). SDH staining was performed with a mixture of 0.2 M sodium succinate (2.5 ml), phosphate-buffered saline (2.5 ml) and nitro-tetranitro blue tetrazolium (5 ml) as described previously[46]. (*B, C*) The number of hair cells per µm (mean ± SD) is presented. A total of 200 cells per area were counted. There were no significant differences from the non-exposure group analyzed by the unpaired t-test. (*D*) Scanning electron microscopy (SEM) for inner hair cells (IHCs) and outer hair cells (OHCs) and (*E*) transmission electron microscopy (TEM) for IHCs at equivalent positions showed no morphological differences in IHCs and OHCs between non-exposed mice (left panels) and WT mice exposed to Mn at 16.50 mg/L via drinking water (Mn, right panels). (*F*) Immunohistochemistry of the SV with anti-KCNQ (Santa Cruz, 1:50), known as one of the marginal cell markers in the SV, and (*G*) TEM for the SV at equivalent positions showed no morphological differences between non-exposed mice (left panels) and WT mice exposed to Mn at 16.50 mg/L via drinking water (Mn, right panels). Scale bars: 100 µm (A), 5 μm (*D*), 2 μm (*E, G*) and 20 μm (*F*).

Figure S2. Schematic summary of Mn-mediated hearing loss with neurodegeneration of spiral ganglion neurons (SGNs) caused by decrease of c-Ret/c-RET protein.

Table S1. Tissue distribution of manganese (Mn) (ng/g wet tissue)

	cerebrum	heart	kidney	muscle	bone	inner ears
control (n=7)	407.5±15	584.8±24	1590.7±42	158.6±18	349.3±5	1069.9±11
Mn (n=7)	379.7±16	568.9±39	1662.6±70	153.5±16	360.1±18	1145.9±26*

After oral administration of Mn at 1.65 mg/L for 2 months, Mn levels (means ± SD) in tissues including the cerebrum, heart, kidney, muscle, bone and inner ears from WT mice exposed to Mn (n = 7) and control mice (n = 7) were measured by inductively coupled plasma-mass spectrometry (ICP-MS) as described previously[41]. Mn levels were calculated by amount of Mn (ng) divided by wet tissue weight (g). Significant difference (*$p < 0.05$) from the control was analyzed by the unpaired t-test.

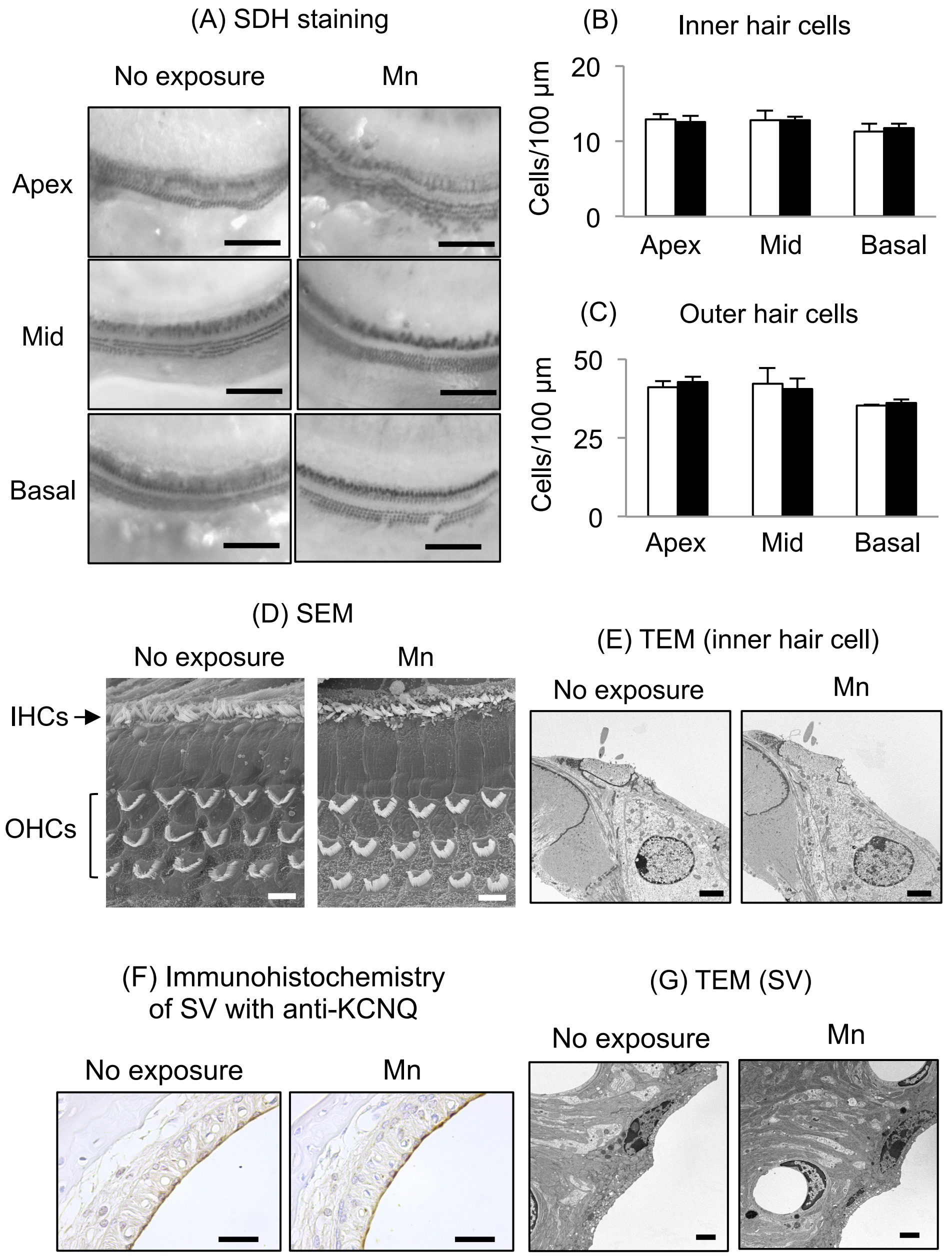

Ohgami et al., Figure S1

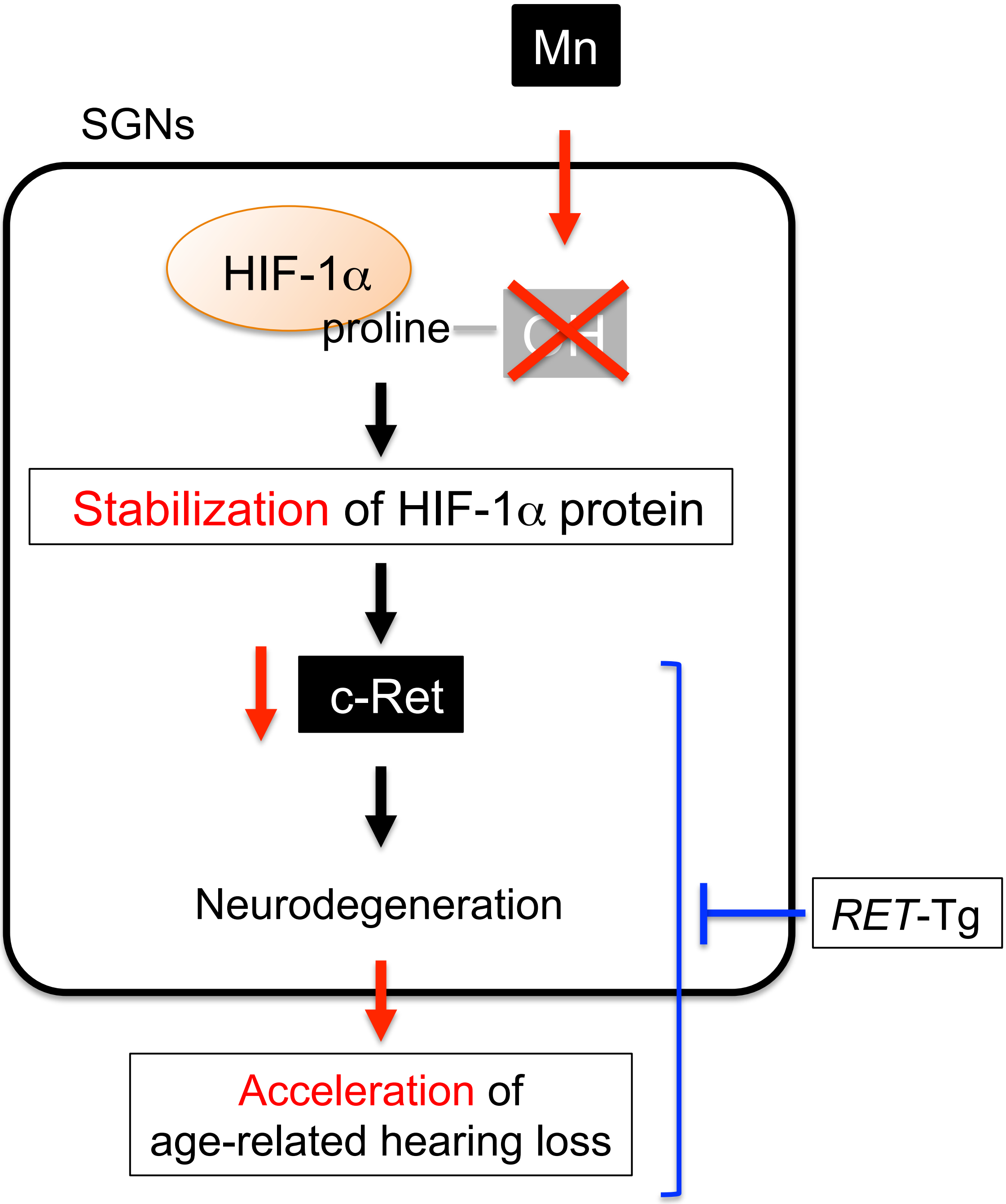

Ohgami et al., Figure S2
